# Circulating Plasma MicroRNAs As Diagnostic Markers for NSCLC

**DOI:** 10.3389/fgene.2016.00193

**Published:** 2016-11-03

**Authors:** Jinpao Hou, Fei Meng, Lawrence W. C. Chan, William C. S. Cho, S. C. Cesar Wong

**Affiliations:** ^1^Department of Health Technology and Informatics, Hong Kong Polytechnic UniversityHong Kong, Hong Kong; ^2^Department of Clinical Oncology, The Queen Elizabeth HospitalKowloon, Hong Kong, Hong Kong

**Keywords:** NSCLC, circulating miRNAs, miRNA panel, diagnostic value, CTCs detection

## Abstract

Lung cancer is the most common cause of cancer deaths all over the world, in which non-small cell lung cancer (NSCLC) accounts for ~85% of cases. It is well known that microRNAs (miRNAs) play a critical role in various cellular processes, mediating post-transcriptional silencing either by mRNA degradation through binding the 3′ UTR of target mRNA or by translational inhibition of the protein. In the past decade, miRNAs have also been increasingly identified in biological fluids such as human serum or plasma known as circulating or cell-free miRNAs, and may function as non-invasive diagnostic markers for various cancer types including NSCLC. Circulating tumor cells (CTCs) are those cells that are shed from solid tumors and then migrate into the circulation. However, reports concerning the roles of CTCs are quite rare, which may be attributed to the difficulties in the enrichment and detection of CTCs in the circulation. Although, there have been reassuring advances in identifying circulating miRNA-panels, which are assumed to be of diagnostic value in NSCLC early stage, some issues remain concerning the reliability of using miRNA panels as a diagnostic tool for NSCLC. In the current review, we are aiming at providing insights into the miRNAs biology, the mechanisms of miRNAs release into the bloodstream, cell-free miRNAs as the diagnostic markers for NSCLC and the current limitations of CTCs as diagnostic markers in NSCLC.

## Introduction

Lung cancer is the most common cause of cancer deaths in men and the second leading cause of cancer deaths in women (Jemal et al., 2010; Ozretić et al., 2012). WHO has shown that lung cancer strikes over 1,605,000 people each year and explains 18.2% of all cancer-related deaths (http://globocan.iarc.fr). There are two types of lung cancer: small cell lung cancer (SCLC) that is a more aggressive type and accounts for 15% of cases, and non-small cell lung cancer (NSCLC), which comprises small squamous cell carcinoma (SCC), adenocarcinoma (AD) and large cell carcinoma (LCC). Remarkably, NSCLC alone accounts for 85% of cases (Travis et al., 2011; Wood et al., 2012). With respect to NSCLC, the main reason for the current high mortality is its late diagnosis and poor prognosis. According to the data from National Cancer Institute, 55% of cases are diagnosed at an advanced stage and over half of the lung cancer patients die within one year of diagnosis (SEER Cancer Statistics Review). In addition, the 5-year survival rates vary from 67% in Stage IA to 39% in Stage IIB, and for patients who were at inoperable advanced stage, the 5-year survival rates even hardly reach 3.3% (Scagliotti et al., 2003; Jemal et al., 2004). Traditional diagnosis of NSCLC tends to be based on either computed tomography (CT) scans or chest X-ray following histological examination of the tissue. Unfortunately, it has been estimated that less than 30% of cases can be detected at an early stage when the curative surgery is possible. The possible reason may be a lack of reliable biological markers indicative of lung cancer at earlier stage when tumors are surgically resectable. Thus far more cases diagnosed are already at an advanced stage when conventional surgical resection of tumor is impractical due to the emergence of tumor metastasis. Furthermore, for various reasons, the tumor tissues tend to be less attainable in clinical practice. Consequently, it is imperative to find a minimally invasive marker for NSCLC early diagnosis, thus improving the prognosis of this disease.

MiRNAs are endogenous small non-coding RNA molecules, 19–22 nucleotides in length, and function as regulatory molecules mediating post-transcriptional silencing either by the promotion of mRNA degradation or by the inhibition of protein translation. The first miRNA was identified in *C. elegans* (Lee et al., 1993). It is well known that miRNAs play a critical role in virtually all signal pathways in various tumor types. With respect to NSCLC, a vast number of reports have shown that the difference between miRNA expression profiles in NSCLC tissues or cell lines and those in healthy controls might be of diagnostic value for NSCLC. For instance, Raponi and colleagues have identified 15 differentially expressed miRNAs between SCCs and healthy lung tissues, among which let-7e and miR-125a were downregulated while the remaining 13 miRNAs were upregulated (Raponi et al., 2009). Similarly, another study by Petriella and coworkers have examined the diagnostic power of miRNAs measurements in fine-needle aspiration NSCLC biopsies and revealed that three miRNAs (miR-7, miR-21, miR-155) exhibited a higher level in tumoral FNA when compared with normal FNA specimens, while let-7a exhibited a lower level (Petriella et al., 2013). Overall, one ambitious aim of these researchers is to develop a reliable miRNA-based method as a convenient tool for the early diagnosis of lung cancer. However, the inevitable invasiveness of using resected tumor samples and the unavailability of tumor tissues limit its routine clinical application. Indeed, not all lung cancer patients have operable diseases and many of them do not have available tumor tissues for genetic analysis (Gao et al., 2011). By contrast, circulating miRNAs in biological fluids such as plasma are emerging as a non-invasive biomarker for NSCLC diagnosis. Most importantly, these non-invasive biomarkers are valuable for predicting drug response by monitoring genetic profiles during treatment, which holds great potential for personalized therapy.

In 2008, 2 research groups reported that human plasma or serum contain a huge amount of miRNAs existing in fairly stable forms, and that these miRNAs profiles hold great promise as novel non-invasive markers for the early diagnosis of cancers (Chen et al., 2008; Mitchell et al., 2008). Since then, reports focusing on the roles of circulating miRNAs in NSCLC have gradually increased, of which the vast majority are involved in determining the cell-free miRNAs panels that can discriminate NSCLCs from healthy controls. Apparently, the analysis of circulating miRNA expression profiles has obvious advantages over that of tissue or cell line-derived miRNAs.

Circulating tumor cells (CTCs), are a group of cells that are shed from solid tumors and then migrate into the circulation. They are widely assumed to be responsible for tumor metastasis (O'Flaherty et al., 2012; Parkinson et al., 2012). Although, CTCs are rare in peripheral circulation, it seems to be attractive to use CTCs as a diagnostic marker of NSCLC, especially when obtaining adequate tissue from patients for diagnosis is difficult. The rarity of CTCs necessitates a more sensitive and more specific detection technique. However, only a few studies reported thus far focus on CTC detection and enumeration, most of which just confirmed the intimate relation between high CTCs numbers and poor prognosis (Hou et al., 2009; Jorge et al., 2012). With regard to CTC-associated miRNAs, only a few studies have been conducted currently to define the subpopulation of CTCs associating it with the prognosis of NSCLC. In the current review, we reviewed miRNAs biology, the mechanisms of miRNAs release into the bloodstream, cell-free miRNAs as diagnostic markers for NSCLC, and the current limitations of CTCs as diagnostic markers in NSCLC.

## miRNA biosynthesis and mode of action

There are mainly two different pathways involved in miRNA biosynthesis: canonical pathway and non-canonical pathway. The canonical pathway involves a step-wise process starting from the nucleus and finishing in the cytoplasm, in which various enzymes and accessory proteins participate. Firstly, long primary miRNA (pri-miRNA) transcript is transcribed by RNA polymerase II (Bartel and Chen, 2004). Then, the resulting pri-miRNA are processed by nuclear RNase III enzyme Drosha and a cofactor Digeorge syndrome critical region 8 (DGCR8), thus producing pre-miRNA of ~70 nt in length (Lund et al., 2004). Subsequently, the nuclear export of pre-miRNA by Exportin-5 into the cytoplasm allows nuclease Dicer cleavage, yielding a paired ~22 nt miRNA/miRNA^*^ duplex. Finally, one strand of the duplex, namely mature miRNA, is loaded into a protein complex containing Argonaute (AGO) to assemble the RNA-induced silencing complex (RISC) responsible for gene silencing, with the remaining strand known as miRNA^*^ undergoing degradation. Afterwards, RISC guided by the mature miRNA binds to the 3′-UTR of target mRNAs resulting in mRNAs degradation or protein synthesis inhibition (Krol et al., 2010). RISC recognizes its target mRNAs by Watson-Crick base pairing between miRNAs and the “seed sequences” of target mRNA sites.

**Figure 1:**
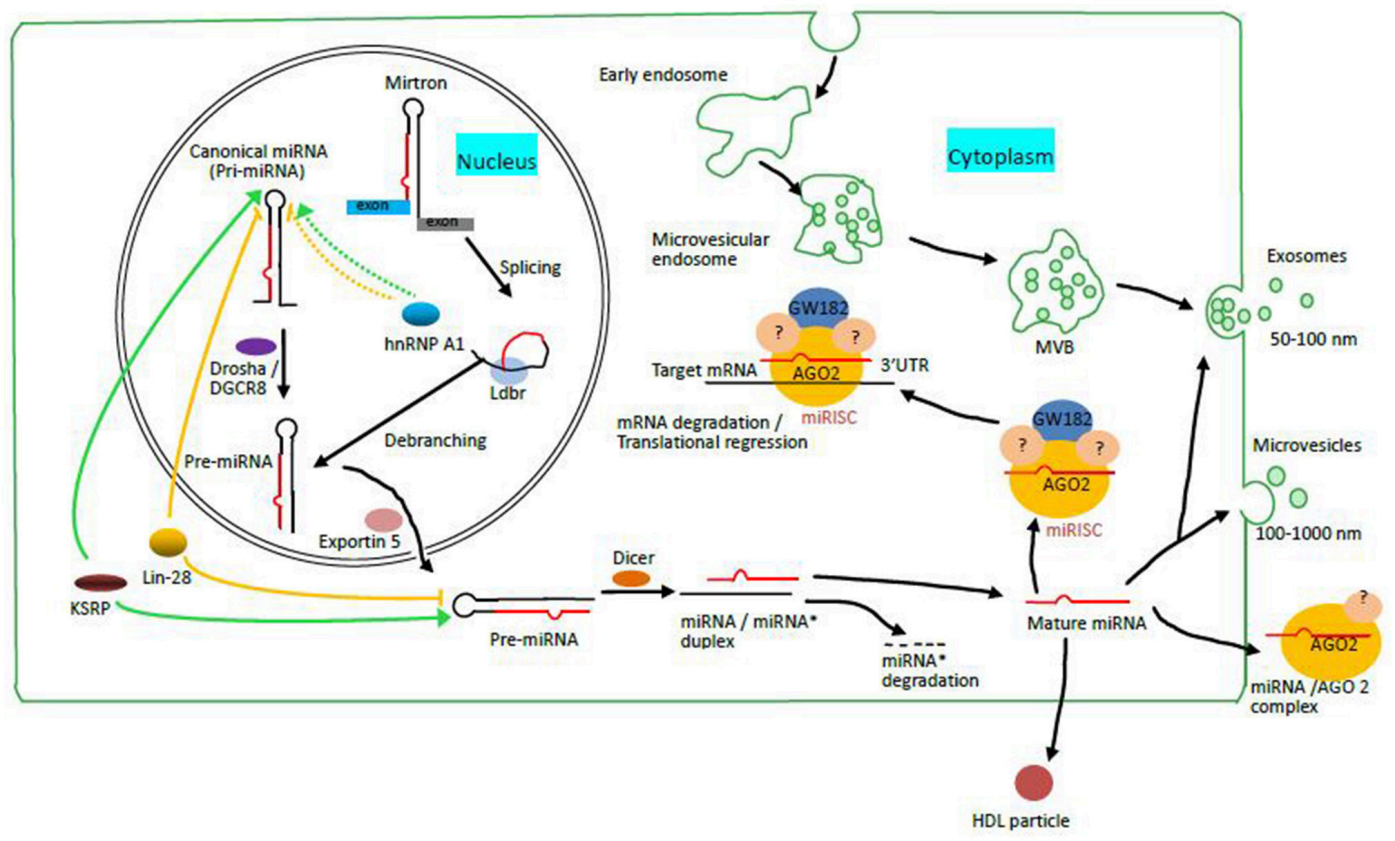
**MiRNA biosynthesis through canonical and non-canonical pathways and the mechanisms of miRNA release into the bloodstream (Modified from Zandberga et al., 2013)**. Mirtrons are spliced, and then debranched by lariat debranching enzyme (Ldbr) to generate pre-miRNAs, the product of Drosha/DGCR 8 cleavage of pri-miRNAs in canonical pathways. Pre-miRNAs from both pathways are exported into the cytoplasm by Exportin 5 for further processing by Dicer, thus producing double-stranded miRNA/miRNA^*^ duplexes. Subsequently, one strand of this duplex is loaded into miRNA induced silencing complex (miRISC) containing one of four AGOs (such as AGO2 as shown), GW182 and various unknown GW182-interacting silencing effectors, leaving the other strand undergoing degradation. The mature miRNA then guides the miRISC to bind to the 3′ UTR of target mRNAs either to promote mRNA degradation or to inhibit protein translation. KSRP as a co-activator and Lin-28 as a co-repressor bind to the terminal loop (TL) elements of pri or pre-miRNAs to promote and inhibit the maturation of a subset of miRNA precursors respectively. HnRNP A1 can both promote (for pri-miR-18a) and inhibit (for pri-let-7a) the processing of miRNAs by binding to the TL elements of pri-miRNAs as shown in the figure, which is mediated by different mechanisms. Circulating miRNAs exist either as a vesicle-associated form or as a protein-associated form in the circulation. The former (vesicles) include exosomes and microvesicles. The latter (proteins) include high and low density lipoproteins, RNA binding proteins such as Argonaute 2.

However, some miRNAs are derived from short hairpin introns called “mirtrons,” the biosynthesis of which involves in a non-canonical pathway. This is a splicing-dependent and Drosha-independent mechanism of miRNA biosynthesis as the primary transcript is spliced and then debranched by lariat debranching enzyme (Ldbr) to form pre-miRNA as a Dicer substrate, which bypasses Drosha/DGCR8 processing (Westholm and Lai, 2011). It is notable that recent studies also have found 5′ and 3′ tailed mirtrons that are very similar to conventional mirtrons. The difference between them is that following debranching, tailed mirtrons contain a single-stranded tail on either 5′ or 3′ of the pre-miRNA-like hairpins, which needs to be trimmed by an exonuclease known as RNA exosome to produce pre-miRNAs for Dicer cleavage (Okamura et al., 2008; Westholm and Lai, 2011). In effect, pre-miRNAs derived from both conventional miRNAs and mirtrons or tailed mirtrons are further exported by Exportin-5 into the cytoplasm, where they are subject to Dicer cleavage to generate miRNA duplex (Figure 1).

Several RNA-binding proteins are involved in a select group of miRNA biosynthesis, acting either as “co-activators” or “co-repressors” for the processing of miRNA precursors. Among them are the three most important proteins: Cell lineage abnormal 28 (Lin-28), KH-type splicing regulatory protein (KSRP) and heterogeneous nuclear ribonucleoprotein A1 (hnRNP A1). Numerous studies have demonstrated that human paralogs Lin-28a and Lin-28b can prevent the maturation of let-7 by interacting with pri- or pre-let-7 via the terminal loop (TL) elements to inhibit their processing by Drosha and Dicer (Choudhury and Michlewski, 2012; Huang, 2012; Mayr and Heinemann, 2013). Intriguingly, Lin-28 protein levels are found to be much higher in undifferentiated cells and the early stages of embryonic development, while mature let-7 miRNAs are absent despite the existence of pre-let-7 (Choudhury and Michlewski, 2012). By contrast, KSRP, also known as FBP2, are shown to act as an auxiliary protein to promote the maturation of select miRNAs including let-7 by binding to TL elements and interacting with Drosha and Dicer, which is similar to the way Lin-28 bind to miRNA precursors (Gherzi et al., 2010). In addition, hnRNP A1 is one of the most abundant members in the protein family of hnRNPs. Unlike lin-28 or KSRP, hnRNP A1 either promotes or inhibits the maturation of miRNAs via different mechanisms according to the miRNA precursor it interacts with. For example, Michlewski G and co-workers showed that hnRNP A1 is required for the processing of pri-miR-18a by binding to its TL element to induce stem-loop structure rearrangement, thus facilitating more effective Drosha cleavage (Michlewski et al., 2008; Choudhury and Michlewski, 2012). However, these authors also have revealed that hnRNP A1 binds to the TL element of pri-let-7a to inhibit Drosha cleavage, thus preventing the processing of pri-let-7a. Most interestingly, they also confirmed the same sequence in the TL element of pri-let-7a is the target of KSRP and proposed that hnRNP A1 and KSRP play antagonistic roles in the maturation of let-7a (Michlewski and Cáceres, 2010). The roles of these protein factors in the processing of miRNA precursors are shown in Figure 1.

## Possible mechanisms of miRNA entering into the circulation

As non-invasive serological markers of tumors, circulating miRNAs are first identified concurrently by two groups in 2008. Mitchell et al. have demonstrated that circulating miRNAs levels in human plasma remain stable even when plasma is subjected to prolonged incubation at room temperature. In addition, they showed that circulating miRNAs are resistant to multiple freeze-thaw cycles. Furthermore, they used a mouse model to reveal that extracellular miRNAs arising from human prostate cancer xenografts enter the circulatory system (Mitchell et al., 2008). By Solexa sequencing, Chen et al. (2008) found that compared to healthy donors, 28 miRNAs species are missing and 63 new miRNA species are detected in lung cancer patients, suggesting that human serum might contain miRNA signatures of the ongoing disease (Chen et al., 2008). These two studies established the concept that one can diagnose cancers based on specific cell-free miRNA signatures. In the meantime, a number of intriguing questions have been posed, among which are the routes of miRNA release into the circulation.

During the past decade, several research groups have found that circulating miRNAs can be packaged into some types of membrane-bound vesicles such as microvesicular bodies (MVB) or exosomes. Given the remarkable stability of circulating miRNA, for several years, the predominant view was that the vast majority of cell-free miRNAs were released from cells in vesicles (Valadi et al., 2007; Zernecke et al., 2009; Collino et al., 2010). However, others have revealed that cell-free miRNAs also exist in vesicle-free forms associating with protein complexes including high and low density lipoproteins and RNA-binding proteins (Wang et al., 2010; Arroyo et al., 2011; Vickers et al., 2011). For instance, Arroyo JD have shown that Argonaute2 (AGO2), the key effector protein of miRNA-mediated silencing, is present in human plasma and serve as a significant carrier of circulating miRNAs in plasma as shown in Figure 1. They also found that potentially 90% of circulating miRNAs are present in vesicle-free form, which suggest that circulating miRNA biomarker analysis based only on exosome purification might be ineffective for miRNA biomarkers that circulate as nonexosomal AGO2 complexes (Arroyo et al., 2011). Additionally, Vicker KC et al. have demonstrated that high-density lipoprotein particles (HDL) transport endogenous miRNAs and deliver them to recipient cells, the process of which is regulated by neutral sphingomyelinase and dependent on scavenger receptor class B type I (Vickers et al., 2011). Moreover, vesicle-associated miRNAs versus vesicle-free miRNAs may originate from different cell types and indicate cell-type specific miRNA release mechanisms. For example, miR-122, a liver-specific miRNA, circulates only in protein-associated form, suggesting that hepatocytes release miR-122 via protein carrier pathway (Chang et al., 2004). By contrast, miRNAs primarily present in membrane-bound vesicles, such as let-7a, may originate from cell types that generate vesicles (Arroyo et al., 2011). However, the relative abundance of vesicle-derived miRNAs versus vesicle-free miRNAs for any given miRNA species remains to be explored.

## Circulating miRNAs as diagnostic biomarkers of NSCLC

The non-invasiveness, stability and reproducibility make circulating miRNAs an ideal diagnostic marker in oncology and hold great promise for clinical applications. Since circulating miRNAs were first identified in human plasma in 2008, a great many of studies have been aiming at identifying a reliable diagnostic tool by investigating the differential expression of circulating miRNAs between NSCLC patients and healthy controls or patients harboring benign tumors. For instance, Lihong Fan et al. have demonstrated that a predictive model combining serum miR-15b-5p, miR-16-5p, miR-20a-5p can be used to discriminate the early stage of NSCLC cases from healthy subjects (Fan et al., 2015). Reassuringly, the fact that this study applied two different methods (RT-qPCR and Nano-quantum dots microarray) to identify the same differential expression of serum miRNAs, which may have improved the sensitivity and reproducibility of the results. Additionally, Li Y et al. have isothermally sensitively detected lung cancer-associated serum miR-486-5p through hairpin probe-based rolling circle amplification (HP-RCA) with superhigh sensitivity (Li et al., 2013). In practice, miR-486-5p has been shown to be downregulated in both the primary cancer and the serum of NSCLC patients in several independent research groups (Volinia et al., 2006; Boeri et al., 2011; Shen et al., 2011), which suggests this miRNA might serve as a tumor suppressor involved in multiple signaling circuits in NSCLC and thus deserves highly attention in future study. Furthermore, a 34-miRNA panel in serum has been identified in patients at the early stage of NSCLC in a group of asymptomatic high-risk subjects with up to 80% accuracy (Bianchi et al., 2011). The signature obtained in this study allows the diagnosis of asymptomatic patients instead of symptomatic ones diagnosed by other signatures in many previous studies. Most importantly, it is specific for lung cancer compared with breast cancer. However, the researchers of this study may well test for the specificity of this signature in other more cancer types other than breast cancer before its clinical application. Moreover, using three different analytical methods, A. Markou and colleagues identified an eight miRNA-panel which can be used for discriminating cancerous tissues from non-cancerous ones in NSCLC patients. By contrast, only three of these miRNAs (miR-30e-5p, miR-21 and miR-10a) are differentially expressed in NSCLC plasma specimens compared to that from healthy plasma, suggesting miRNA profiles in the circulation may not necessarily reflect exactly those in tumor tissues (Markou et al., 2013). However, an earlier report have showed that miRNA footprints did not show significant difference between circulating exosome-derived miRNAs and the tumoral miRNAs profiles using 4 lung cancer tissues and their corresponding plasma samples (Rabinowits et al., 2009). This contradiction between these two studies may be attributed to two factors: one is that the internal control gene used for the normalization of miRNA expression may vary between different research groups; the other is that circulating miRNAs do not exist only in exosomes as mentioned earlier, most of them are shown to be associated with protein complexes. Most importantly, only 4 lung cancer tissues used for the study are not enough to represent a reliable result.

In the past two years, a vast number of efforts have been made to identify circulating miRNA panels for NSCLC early diagnosis. However, many of these studies remain to be validated given the inconsistency of reference controls used for the normalization and of the materials such as exosomes, serum and whole blood used for RNA isolation. For instance, Magdalena B. Wozniak and colleagues showed that a 24-miRNA panel alone could distinguish lung cancer patients from healthy controls with an AUC of 0.92, and they also revealed that this diagnostic power can be further enhanced by adding factors such as age, sex and smoking status into this model (Wozniak et al., 2015). Similarly, 6 cell-free miRNAs (200b, 429, 205, 125b, 34b, 203) have also been validated with an even higher abundance in the serum of NSCLC patients compared to healthy subjects (Halvorsen et al., 2016). However, a common problem in these two studies is that the authors have chosen U6 as the internal control for the normalization when analyzing the miRNAs profiles, which may be inaccurate as U6 has been reported to be not a suitable endogenous control for the quantification of cell-free miRNAs (Benz et al., 2013; Xiang et al., 2014). Therefore, it is conceivable that the conclusions obtained from those studies should be interpreted with caution. In addition, Silva et.al reported that the levels miR-30e-3p, let-7f and miR-20b, were downregulated in the plasma vesicles of NSCLC cases compared with healthy controls (Silva et al., 2011). Another similar study showed that a panel of miRNAs (30b, 30c, 103, 122, 195, 203, 221, 222) in exosomes from the plasma displayed significantly differential expression between NSCLC cases and healthy donors, which might suggest the possibility that miRNAs signatures from patient exosomes could alone represent the status of ongoing disease (Giallombardo et al., 2016). However, these studies might not be complete as researchers focused only on vesicle-associated miRNAs, such as exosomes, probably missing the differential expression of vesicle-free miRNAs that may represent a larger proportion of miRNAs in the circulation and thus producing an inaccurate result. Most of the reported results identifying circulating miRNA panels as diagnostic biomarkers in the last two years are summarized in following Table 1. As shown in the table, different miRNA-panels have been identified by different research groups. Actually, some above mentioned factors such as various biological materials and different internal controls used may account for this phenomenon.

**Table 1:** **Circulating miRNAs as diagnostic markers for NSCLC**.

**Significantly-expressed miNRAs**	**Scope**	**Sample**	**Technique**	**Normalization**	**References**
let-7c, miR-152 (down)	120 NSCLCs vs. 360 HCs	Plasma	qRT-PCR	U6	Dou et al., 2015
miR-16-5p, miR-17b-5p, miR-19-3p, miR-20a-5p, miR-92-3p (down) miR-15b-5p(up)	Training set: 94 NSCLCs vs. 58HCs, Validation set: 70 NSCLCs vs. 54 HCs	Serum	TaqMan miRNA assays	Absolute quantification	Fan et al., 2015
miR-148/152 family (Down) miR-944, miR-3662 (up) miR-483-5p, miR-193a-3p, miR-25, miR-214 miR-7 (up)	20 NSCLCs with BPD VS.10 HCs 90 NSCLCs vs. 85 HCs 221 NSCLCs, 161 HCs, 56 with benign nodules	Serum Plasma	RT-qPCR RT-qPCR TaqMan Low Density Array, RT-qPCR	U6 U6 let-7d/g/i trio	Chen et al., 2015; Li et al., 2015; Powrózek et al., 2015
miR-125a-5p, miR-145 miR-146a (up)	70 NSCLCs vs. 70 HCs	Serum	RT-qPCR	miR-39	Wang et al., 2015
A panel of 24 miRNAs (Relative expression)	100 NSCLCs vs. 100 HCs	Plasma	TaqMan MiRNA Arrays	U6snRNA ath-miR-159a	Wozniak et al., 2015
A panel of 8 miRNAs (Relative expression)	12 NSCLCs vs. 6 HCs	Exosomes	RT-PCR	mir-1228-3p	Giallombardo et al., 2016
miR-429, miR-205, miR-200b, miR-203, miR-125b miR-34b (up)	38 NSCLCs, 16 patients with COPD, 16 HCs	Serum	TaqMan Low Density Arrays	U6	Halvorsen et al., 2016

NSCLCs, NSCLC patients; HCs, healthy controls; RT-PCR, real-time reverse transcription quantitative polymerase chain reaction; COPD, chronic obstructive pulmonary disease; BPD, benign pulmonary diseases.

Although, there do exist some concerns about current studies of circulating miRNAs, some critical miRNAs as tumor suppressors or oncogenes are of great value to develop miRNA-based strategy for lung cancer therapy and therefore should be prioritized to further studies with respect to their diagnostic power. Among these critical miRNAs, miR-21 is a known to be an “oncomir” and is commonly overexpressed in cancers including lung, breast and colorectal cancer (Ma et al., 2014). For example, Si ML and colleagues showed that miR-21 inhibition suppresses tumor growth in xenograft mouse model and increases cell apoptosis that is associated with downregulation of bcl-2 expression in breast cancer (Si et al., 2007). Also, higher expression of miR-21 and miR-155 in both tumor tissues and serum can predict recurrence and poor survival in NSCLC (Yang et al., 2013). This may suggest that overexpression of circulating miR-21 holds great prognostic potential in NSCLC. Another aspect about miR-21 is that it influences response to chemotherapy in several tumor types and thus can act as a therapeutic target for overcoming drug resistance in cancers. For instance, a recent study has revealed that antisense inhibition of miR-21 or miR-221 sensitizes the effects of Gemcitabine, a chemotherapeutic treatment of pancreatic cancer (Park et al., 2009). By contrast, let-7 family miRNAs are known as tumor suppressors and down-regulation of let-7 expression has been demonstrated in breast, prostrate, ovarian and lung cancers (Boyerinas et al., 2010). Dejuan Kong et al. have provided evidence that let-7 loss mediates up-regulation of EZH2 and contributes to PCa aggressiveness, which can be attenuated by B-DIM, a potent agent in inhibiting the growth of Pca cells (Kong et al., 2012). In addition, numerous studies have revealed that let-7 acts as a promising therapeutic target for lung cancer. For example, Trang.s group have shown that systematical delivery of synthetic let-7 mimic or miR-34a into lung tumor-bearing mice results in significant tumor regression compared to the delivery of a miRNA control (Trang et al., 2011). Overall, since current studies on the roles of miR-21 and let-7 family in various cancer types have mainly focused on tumor tissues, circulating miR-21 and let-7 in plasma warrant further validation before applying them as biomarkers for the early diagnosis of cancers including NSCLC.

## Current limitations on CTCs as diagnostic markers of NSCLC

CTCs are tumors cells which are released from solid tumors, and then migrate into the circulation and play a critical role in tumor metastasis. However, CTCs are extremely rare, and they can be detected at a low frequency of 1 CTCs per 10^6^-10^7^ leukocytes (Young et al., 2012). Initially, CTCs were considered as non-leukocytic cells with epithelial origin since they have been detected in various epithelial cancers such as breast cancer, lung cancer and colon cancer (Allard et al., 2004). Consequently, epithelial marker-based methods for the detection of CTCs have been widely used (Hardingham et al., 1993; Liljefors et al., 2005). Nevertheless, recent studies have shown that the morphological characteristics of CTCs have yet to be well defined and may vary according to tumor stages (Parkinson et al., 2012). Overall, more clear phenotypes of CTCs as a “liquid biopsy” remain to be explored in future study.

As a “liquid biopsy,” current studies on CTCs as a biomarker of NSCLC have been limited to its enrichment and detection as well as its relation to NSCLC prognosis. For instance, Laura Muinelo-Romay and colleagues found that patients with CTCs >5 are assumed to correlate with poorer prognosis based on CTCs counts using CellSearch technology (Muinelo-Romay et al., 2014). Another group have isolated CTCs by cell size filtration and shown that CTCs can be identified in two-thirds of patients at advanced stage of NSCLC at diagnosis, suggesting that CTCs counts may reflect the ongoing disease status of NSCLC (Mascalchi et al., 2016).

However, less progress has been made in the field of CTCs as an ideal biomarker for NSCLC diagnosis, and this may result from several aspects. For one thing, various techniques for CTCs enrichment and later detection have been developed which base either on physical properties such as size, density and electric charges, or on biological properties such as the expression of CTCs surface marker (Alix-Panabieres and Pantel, 2013), among which the most widely used method relies on the epithelial protein, namely, Epithelial cell adhesion molecule (Ep-CAM) for the CTCs enrichment (Allard et al., 2004; Harb et al., 2013; Haus et al., 2014). However, Hanssen A et al. reported that the expression Ep-CAM may be downregulated in CTCs experiencing epithelial-to-mesenchymal transition (EMT), which raises the concern that this Ep-CAM based CTCs enrichment method may lose EMT-associated CTCs (Hanssen et al., 2016). Additionally, another group, Yingchun Man et.al have developed four CTCs markers (CK7, CLCA2, HMMR and hTERT) to detect CTCs, which significantly improves the specificity and sensitivity of CTC detection (Man et al., 2014). Intriguingly, by miRNA in situ hybridization, Francisco G. Ortega et al. have identified miR-21 within CTCs as a marker used for detecting CTCs displaying an EMT phenotype (Ortega et al., 2015). We can infer that miR-21 may be function as an oncomir implicated in tumor metastasis. In a nutshell, whether epithelial maker-dependent or -independent approach is more sensitive and specific in CTCs enrichment and detection remains to be explored. For another thing, CTCs counts alone may not fully represent the ongoing NSCLC status especially at a very early stage. This concept is clearly intelligible given that several results have shown CTCs heterogeneity as a diagnostic marker by identifying different CTCs subpopulations. In a research reported by Wu et al. (2015), three CTCs subpopulations, epithelial CTCs, biophenotypic epithelial/mesenchymal CTCs and mesenchymal CTCs, were identified using epithelial-to-mesenchymal transition markers, which is useful to identify the more aggressive CTC phenotypes and thus facilitates the determination of clinical practice.

## Discussion

NSCLC diagnosis, especially at early stage is in great demand in clinical practice. Conventional diagnostic applications relying on tissue biopsy either through computed tomography scans or chest X-ray have remarkable limitations given their low sensitivity and invasiveness as well as tissue unavailability. Consequently, a novel, reliable and reproducible diagnostic biomarker is badly needed to obtain an earlier and more accurate diagnosis result.

Circulating miRNAs in biological fluids, especially in human plasma or serum, have emerged as a non-invasive diagnostic marker for NSCLC. In the past five years, studies in this field have identified different miRNA panels in the circulation with higher sensitivity plus higher specificity in early NSCLC diagnosis, which might be preferable compared to any single miRNA used for NSCLC diagnosis. In addition, several factors may contribute to the fact that different miRNA panels have been identified for NSCLC diagnosis. To begin with, different materials such as plasma/serum, whole blood, or exosomes used for cell-free miRNAs isolation make it possible that the quality and quantity of circulating miRNAs isolated by different researcher vary according to biological material types. Indeed, whole blood also contains a large amount of peripheral blood mononuclear cells (PBMCs) compared with serum or plasma. Intriguingly, a two-miRNA panel (miR-19b-3p and miR-29b-3p) arising from PBMCs have been developed by Jie Ma et al. to discriminate NSCLCs from healthy persons with 72.62% sensitivity and 82.61% specificity. This group also identified SCC, the major type of NSCLC using this two-miRNA panel, with 80.00% sensitivity and 89.86% specificity, which may imply that only focusing on serum-derived circulating miRNAs may not be enough to reflect a reliable result in terms of NSCLC diagnosis based on circulating miRNA detection (Ma et al., 2015). In addition, the inconsistency of the methods used for data analysis should also be considered to account for the existence of various miRNA-panels. As shown in Table 1, internal controls such as U6 and miR-39 have been used for the normalization of miRNA expression. Unfortunately, the high variability of non-coding RNAs including rRNAs, U6 snRNA and snoRNAs in the plasma microvesicles and their readily degradation in serum have been reported, which indicates that such non-coding RNAs may be inappropriate as endogenous controls (Chen et al., 2008). On a whole, more validations on the whole blood are warranted to identify circulating miRNA biomarkers that are of diagnostic value, coupled with a uniform and reliable internal control for data analysis.

CTCs are another type of “liquid biopsy” ideal for the diagnosis of NSCLC. Several issues remain to be addressed with respect to CTC enrichment and detection. Firstly, the platform of CTC detection technology combining marker-dependent (Cell Search) and marker-independent approaches (Isolation by size of epithelial tumor cells) has proven to be more sensitive than either of the two methods alone could be (Krebs et al., 2012). In addition, CTC-associated miRNAs, such as miR-21, can be applied to detect CTCs with an EMT phenotype (Ortega et al., 2015). We may hypothesize here CTC-associated miRNAs are likely to contain a larger amount of information underlying NSCLC cases, not merely being used as a marker for detection of CTCs. On the whole, future validation about such work is warranted before routine clinical applications are available.

## Author contributions

LC, WC, and SW: Contributed the research concept and design; Moreover, LC: Finished critical revision of this article; JH: Finished the collection and assembly of data, and then she wrote the article; FM: Finished the drawing of the figure in this article.

### Conflict of interest statement

The authors declare that the research was conducted in the absence of any commercial or financial relationships that could be construed as a potential conflict of interest.

## Abbreviations

MVB: Microvesicular bodies
AGO: Argonaute proteins
MiRISC: miRNA-induced silencing complex
3′ UTR: 3′untranslated region
Ldbr: lariat debranching enzyme
KSRP: KH-type splicing regulatory protein
hnRNP A1: heterogeneous nuclear ribonucleoprotein A1.
